# Complex interplay of gut microbiota between obesity and asthma in children

**DOI:** 10.3389/fmicb.2023.1264356

**Published:** 2023-11-03

**Authors:** Mingge Hu, Xiaoman Zhao, Yazun Liu, Huan Zhou, Yannan You, Zheng Xue

**Affiliations:** Shanghai Municipal Hospital of Traditional Chinese Medicine, Shanghai University of Traditional Chinese Medicine, Shanghai, China

**Keywords:** gut microbiota, obesity, asthma, children, lipid metabolism, chronic low-grade inflammation, immune

## Abstract

Obesity is an important risk factor and common comorbidity of childhood asthma. Simultaneously, obesity-related asthma, a distinct asthma phenotype, has attracted significant attention owing to its association with more severe clinical manifestations, poorer disease control, and reduced quality of life. The establishment of the gut microbiota during early life is essential for maintaining metabolic balance and fostering the development of the immune system in children. Microbial dysbiosis influences host lipid metabolism, triggers chronic low-grade inflammation, and affects immune responses. It is intimately linked to the susceptibility to childhood obesity and asthma and plays a potentially crucial transitional role in the progression of obesity-related asthma. This review article summarizes the latest research on the interplay between asthma and obesity, with a particular focus on the mediating role of gut microbiota in the pathogenesis of obesity-related asthma. This study aims to provide valuable insight to enhance our understanding of this condition and offer preliminary evidence to support the development of therapeutic interventions.

## 1. Introduction

Asthma and obesity are important public health concerns affecting children's health. According to data from the 2021 Global Asthma Network Stage I cross-sectional study, approximately 9.1% of children and 11% of adolescents had asthma in the previous year, with nearly half experiencing severe symptoms (Asher et al., 2021). In 2016, the worldwide prevalence of obesity among children and adolescents in boys and girls aged 5–19 years were 7.8 and 5.6%, respectively (Bentham et al., 2017). An estimated 206 million children and adolescents will experience obesity worldwide by 2025; this number is expected to reach 254 million by 2030 (Jebeile et al., 2022). Obesity and asthma are not simply coexisting conditions; research indicates that obese children have a >50% higher risk of developing asthma than normal-weight children (Malden et al., 2021). In 2017, the Centers for Disease Control and Prevention identified obesity as a significant risk factor for asthma (Grossman et al., 2017).

The increased risk of childhood asthma due to obesity may be attributed to early-life experiences or parental factors. Notably, rapid weight gain during the initial 6–18 months after birth is strongly linked to a 2.1–3.3 times higher risk of non-atopic asthma; this correlation is particularly pronounced among boys (Ho et al., 2022). Furthermore, there is a linear relationship between the risk of childhood asthma and an increase in maternal pre-pregnancy body mass index (BMI) (Rosenquist et al., 2023). Clinical symptoms tend to be more severe in children with comorbid asthma and obesity, who experience more frequent exacerbations. Additionally, disease control is often poorer in this group and characterized by reduced responsiveness to inhaled corticosteroids and an increased likelihood of unresponsiveness to bronchodilators (Peters et al., 2018). In 2014, the Global Initiative for Asthma identified “asthma with obesity” as an asthmatic phenotype (Reddel et al., 2015). The above evidence suggests a correlation between asthma and obesity and that obesity-related asthma has specific mechanisms and treatments.

Obesity complicates asthma phenotypes, and previous research highlighted its possibility associated with obesity-induced lipid status disturbances and chronic systemic inflammation (Miethe et al., 2020). In addition to classical atopic asthma, children with obesity-related asthma exhibit CD4^+^ T cells polarizing toward Th1 and Th17 profiles (Nyambuya et al., 2020; Leija-Mart́ınez et al., 2022). With technological advancements in the microbiome field, increasing evidence has linked obesity, asthma, and dysbiosis of the gut microbiota. The human body consists of trillions of microbes that congregate in the intestine to form a complex community known as the gut microbiota (Adak and Khan, 2019). The gut microbiota is a complicated and dynamic ecosystem that coevolves with the host, develops during infancy, and plateaus during adulthood (Bäckhed et al., 2015). It plays a role in regulating host lipid metabolism and the inflammatory response as well as stimulating the development of the immune system by assisting the host in digesting food and releasing nutrients (Yu et al., 2019; Zhuang et al., 2019). The disruption of healthy and timely microbial colonization has long-term health effects, particularly increased susceptibility to allergic and metabolic diseases (Lloyd and Marsland, 2017; Zhang and Dang, 2022). Increasing evidence suggests that the gut microbiota may play a bridging role in the mechanisms underlying the increased risk of obesity and asthma. However, this series of complex mechanism changes has yet to be fully elucidated and interconnected. This article summarizes the latest research on the correlation between obesity and asthma and provides a detailed explanation of the potential mediating mechanisms of the gut microbiota.

## 2. Impact of early-life gut microbiota colonization on asthma and obesity

### 2.1. Factors influencing early-life gut microbiota colonization

**Figure 1:**
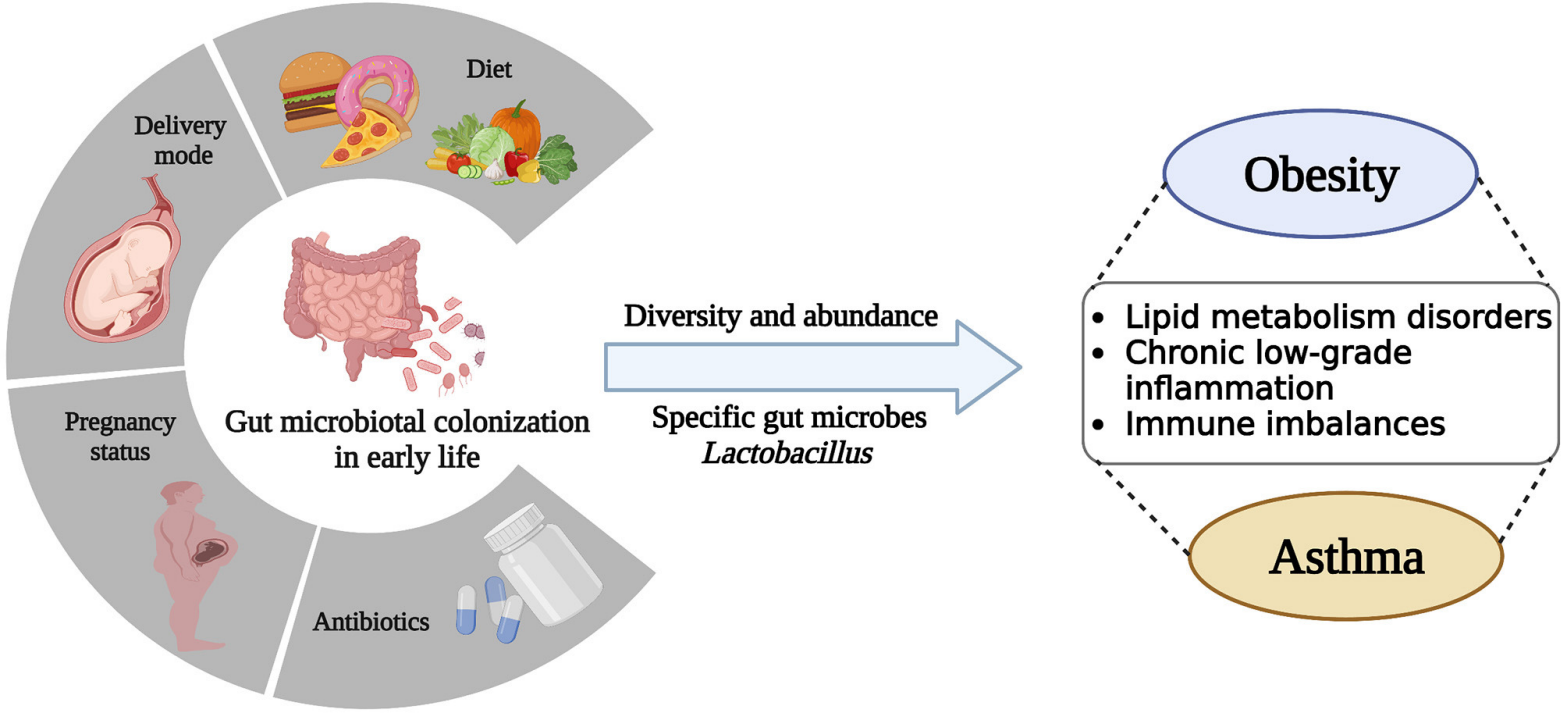
Early-life gut microbiota colonization is disturbed by a variety of factors, including pregnancy status, delivery mode, diet, and antibiotic treatment, leading to alterations in microbial diversity, abundance, and specific microbes, which ultimately contribute to dysfunction and disease in the host.

The postnatal period is often referred to as the “window of opportunity,” a critical time for microbial colonization as well as the rapid maturation and development of various systems in children, including the immune and metabolic systems (Johnson and DePaolo, 2017; Robertson et al., 2019). These systems evolve in tandem and are highly interdependent, strongly supporting children's growth. Maternal pregnancy status, delivery mode, diet, and early-life antibiotic treatment are important factors that influence gut microbial colonization and development (Gibson et al., 2015; Wu et al., 2016a; Lundgren et al., 2018) (Figure 1).

Whether the fetus environment in the womb is sterile remains inconclusive; however, scholars concur that the mother's nutritional and immune inflammatory state during pregnancy can affect the offspring's growth and development and that the gut microbiota potentially plays a mediating role in this process (Chu et al., 2016; Theis et al., 2019). A study reported that the placentas of women who experience excessive weight gain during pregnancy and preterm delivery are characterized by an increased abundance of *Firmicutes, Actinobacteria*, and *Cyanobacteria* and a decreased abundance of *Proteobacteria* (Antony et al., 2015).

Mode of delivery affects the neonatal gut microbiota. The microbiota of newborns delivered vaginally closely resembles that of the mother's birth canal, whereas that of newborns delivered via cesarean section closely resembles that of the mother's skin. The guts of newborns delivered vaginally is predominantly populated by *Lactobacillus, Prevotella, Atopobium*, and *Sneathia* spp., whereas that of newborns delivered via cesarean section is predominantly populated by *Staphylococcus, Corynebacterium*, and *Propionibacterium* spp. with delayed colonization by the *Bifidobacterium* and *Bacteroides* genera (Dominguez-Bello et al., 2010; Jakobsson et al., 2014; Rutayisire et al., 2016).

Diet influences the composition and function of the gut microbiota. Compared to formula feeding, breastfeeding increases the diversity of gut microbiota species and alters the levels of specific bacterial genera by increasing the abundance of *Bifidobacterium* spp. and decreasing the abundance of *Clostridium* spp. and *Bacteroides* spp. (Savage et al., 2018). Changes in dietary patterns shape the gut microbiota of children as they age; these changes occur over a short period (David et al., 2014). One study reported that a high-fat diet (HFD) led to a decrease in microbial populations, alterations in species abundance, and increased in intestinal permeability (Turnbaugh et al., 2009). A low-fat diet decreases the relative abundance of *Actinobacteria* and *Firmicutes*, whereas a low-carbohydrate diet increases the relative abundance of *Proteobacteria, Bacteroidetes*, and *Firmicutes* phyla (Fragiadakis et al., 2020).

Antibiotic administration eliminates antibiotic-sensitive bacteria and reduces the abundance and diversity of the gut microbiota in children. A previous study reported that azithromycin exposure reduces microbiota alpha diversity (McDonnell et al., 2021). It takes approximately 1 month for microbial diversity to recover after antibiotic administration in children (Yassour et al., 2016). However, exposure to antibiotics may increase the total microbial load in the gut by eliminating of sensitive bacteria and increasing in the reproduction of antibiotic-resistant microbiota (Panda et al., 2014; Liu et al., 2021). Furthermore, the inappropriate use of antibiotics can stimulate bacterial resistance, which can be transferred from the mother to the newborn (Karami et al., 2006).

### 2.2. Gut microbiota dysbiosis increases risks of childhood obesity and asthma

Delayed maturation and inappropriate development of the microbiome can disrupt the host's normal growth trajectory, leading to overnutrition and an immune imbalance (Gensollen and Blumberg, 2017). Numerous clinical studies and epidemiological data have consistently indicated that alterations in the diversity and specific species of the gut microbiota are associated with childhood obesity and asthma (Zhang et al., 2015; Garcia-Larsen et al., 2016). The mother's dietary pattern during pregnancy can influence the infant's gut microbiot; this exposure factor may affect the offspring's risk of asthma (Alsharairi, 2020). Breastfeeding increases the diversity of the gut microbiota in infants, which protects against childhood asthma and obesity (Oddy, 2017; Forbes et al., 2018). Children born via cesarean section are more prone to developing asthma and obesity than those born vaginally owing to disrupted gut microbiota colonization patterns; this effect continues during adolescence and adulthood (Yuan et al., 2016; Gürdeniz et al., 2022). Repeated exposure to antibiotics before 6 months of age is associated with weight gain during childhood (Saari et al., 2015). As antibiotic prescriptions decrease and the gut microbiota is protected, the incidence of childhood asthma has declined in certain regions of Europe and North America (Patrick et al., 2020).

An increased ratio of *Firmicutes* to *Bacteroidetes* is a marker of gut microbial dysbiosis in obese children (Bervoets et al., 2013). The fecal microbiota of ob/ob and HFD-induced obese mice showed an increased abundance of *Firmicutes* and decreased abundance of *Bacteroidetes* (Ley et al., 2005; Jo et al., 2021). *Firmicutes* may mediate susceptibility to overweight/obesity during pregnancy and in offspring aged 1–3 years (Tun et al., 2018). A clinical trial reported a 20% increase in *Firmicutes* abundance resulting in a 150-kcal increase in energy absorption (Jumpertz et al., 2011). This suggests that a microbiota dominated by *Firmicutes* exhibits a higher energy extraction efficiency than that dominated by *Bacteroidetes*. Compared with the gut microbiota of their lean littermates, the gut microbiota of obese ob/ob mice is characterized by a higher abundance of indigestible dietary polysaccharides, such as starch, sucrose, and galactose, as well as Mollicutes, suggesting that obese ob/ob mice have a greater capacity to extract energy from food (Turnbaugh et al., 2006, 2008).

Several specific gut microbiota are correlated with asthma. Lower *Bifidobacterium* and *Akkermansia* loads and higher *Candida* and *Rhodotorula* loads are reportedly associated with atopic asthma in children (Fujimura et al., 2016). Significantly lower relative abundances of *Lachnospira, Veillonella, Faecalibacterium*, and *Rothia* genera early in life place infants at risk of later developing asthma (Arrieta et al., 2015). Probiotic intervention protects against allergic diseases in infants delivered via cesarean section who are at high risk of allergies; this beneficial effect was similarly observed in mice (Hogenkamp et al., 2015; Kallio et al., 2019). *Lactobacillus* supplementation ameliorates clinical symptoms in children with asthma, whereas *Bifidobacterium* supplementation reduces neutrophil and eosinophil infiltration in severely asthmatic mice (Huang et al., 2018b; Raftis et al., 2018).

*Lactobacillus* species may be involved in obesity-related asthma. Supplementation with *Lactobacillus* reportedly reduces airway inflammation and asthma symptoms in school-aged children while restoring anti-inflammatory fatty acid (FA) metabolites in infants at high risk for asthma (Chen et al., 2010; Durack et al., 2018). In obese individuals, treatment with *Lactobacillus* ameliorates body weight and reduces fat levels (Kadooka et al., 2010; Crovesy et al., 2017). Moreover, it significantly reduces adipose tissue accumulation in HFD-induced obese mice (Lee et al., 2021). In a recent study of asthma in obese mice, nitro-oleic acid treatment reduced lung and total respiratory elasticity, which has been associated with elevated *Lactobacillus* abundance (Heinrich et al., 2023). This study suggests a relationship between *Lactobacillus* and obesity-related asthma; however, the specific mechanisms involved require further investigation.

## 3. Mediating role of gut microbiota between childhood obesity and asthma

### 3.1. Lipid metabolism

#### 3.1.1. Obesity-accompanied dysregulated lipid metabolism contributes to asthma development

Lipids are crucial components and energy reserves of cells with significant regulatory functions in metabolism, inflammation, immunity, and various other pathways. Lipid metabolism disorders are considered primary pathological factors contributing to obesity. Patients with asthma exhibit significant alterations in phospholipid and sphingolipid levels, suggesting that abnormalities in lipid metabolism are involved in asthma development (Murphy et al., 2021; Rago et al., 2021). Adenosine monophosphate activated protein kinase (AMPK) is a key regulator of lipid metabolic balance *in vivo* (Herzig and Shaw, 2018). AMPK suppresses the process of *de novo* fatty acid synthesis (FAS) by causing inhibitory phosphorylation of Acetyl-CoA Carboxylase 1 (ACC1) (Jeon, 2016). Simultaneously, it promotes fatty acid oxidation (FAO) by facilitating inhibitory phosphorylation of ACC2, leading to the activation of Carnitine Palmitoyltransferase-1 (CPT-1) activity (Fang et al., 2022). AMPK levels are negatively correlated with obesity and asthma (Herzig and Shaw, 2018; Garcia et al., 2019). The AMPK pathway was inhibited in an obesity-related asthma model constructed through ovalbumin sensitization and stimulation; however, AMPK activation alleviated both airway inflammation and airway hyper-responsiveness (AHR) in a mouse model (Zhu et al., 2019).

Lipid mediators produced via arachidonic acid (AA) pathway influence asthma (Monga et al., 2020). AA is stored in membrane phospholipids and released by phospholipase A2 (PLA2) upon exposure to allergens (Wang et al., 2021). PLA2 induces the enzymatic and non-enzymatic oxidation of AA to prostaglandins, leukotrienes, and other bioactive mediators, exerting receptor-specific stimulatory and inhibitory effects that influence the pathophysiology of asthma (Samuchiwal and Boyce, 2018). Increased protein expression of sPLA2-X in the airway epithelial cells of patients with asthma is associated with AHR (Hallstrand et al., 2013). In asthma models, prostaglandins E_2_ reduces lung inflammation and remodeling, showing beneficial effects in asthma patients (Insuela et al., 2020).

The adipocytokines leptin and adiponectin regulate lipid metabolism by influencing appetite. Leptin inhibits orexic neurons and stimulates anorexic proleptin neurons to regulate appetite (Obradovic et al., 2021). Adiponectin, on the other hand, increases during fasting, and activates the AMPK pathway by binding to its receptor AdipoR1 (Okada-Iwabu et al., 2013). In the adipose tissue of individuals with obesity, adipocyte cytokine leptin levels increase, whereas adiponectin levels decrease (Frithioff-Bøjsøe et al., 2020). Both adipocytokines and their receptors are expressed in human lungs and are associated with asthma severity in children. Leptin levels are positively correlated with the prevalence and severity of childhood asthma, whereas adiponectin levels are negatively correlated, particularly in boys (Assad and Sood, 2012).

#### 3.1.2. Gut microbiota regulates asthma lipid metabolism: the key role of SCFAs

The impact of the gut microbiota on host lipid metabolism has been extensively demonstrated in both human and animal models. Implementing energy restriction and dietary interventions in obese individuals can increase the microbiota gene abundance while simultaneously reducing blood lipid levels (Cotillard et al., 2013). Conventional mice had a 60% higher body fat content and insulin resistance level than germ-free (GF) mice (Bäckhed et al., 2004). GF mice exhibited HFD-induced insulin resistance and improved cholesterol metabolism, which might be related to an increase in FAO in the peripheral tissues owing to enhanced AMPK activity *in vivo* (Rabot et al., 2010). The transplantation of gut microbiota from ob/ob mice into GF mice resulted in a notable increase in both body weight and body fat content (Turnbaugh et al., 2006). Young mice treated with antibiotics showed an altered gut microbiota composition and elevated levels of hormones related to carbohydrate, lipid, and cholesterol metabolism (Cho et al., 2012).

**Figure 2:**
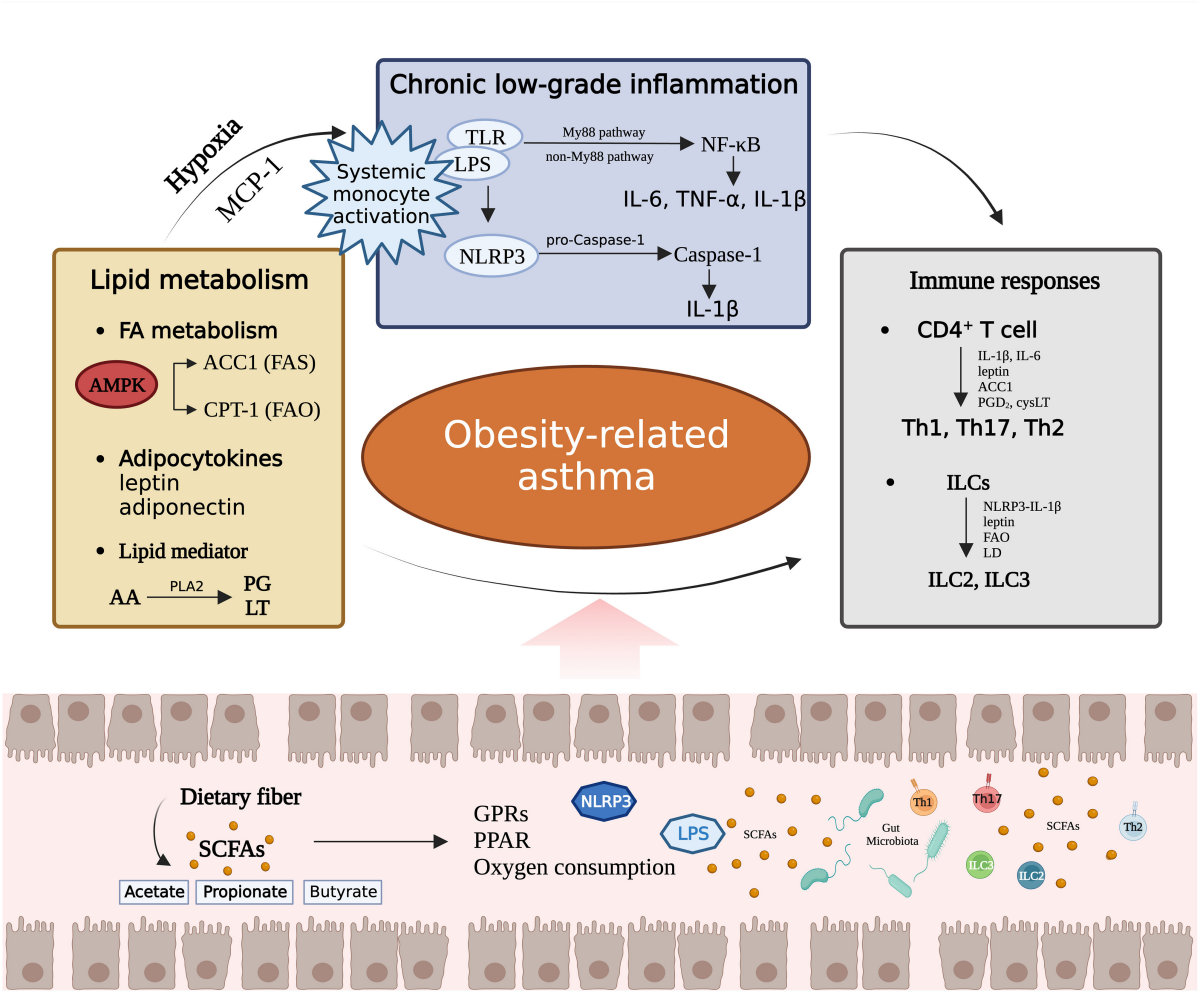
The effects of the gut microbiota on lipid metabolism, chronic low-grade inflammation, and immunity, as well as the interactions among the three pathways. PG, prostaglandin; LT, leukotriene; MCP-1, Monocyte chemoattractant protein 1; PGD_2_, prostaglandin D_2_; cysLT, cysteinyl leukotriene; LD, Lipid droplet.

Short-chain FAs (SCFAs), such as acetate, propionate and butyrate, are the final products of microbial fermentation (Ŕıos-Covián et al., 2016; Agus et al., 2021). SCFAs provide substrates for lipid synthesis and serve as regulatory factors to modulate lipid metabolism in both brown and white adipose tissues (Gao et al., 2009; Li et al., 2018; He et al., 2020). SCFAs regulate host biological processes via ligand receptor interactions with G protein-coupled receptors (GPRs), while peroxisome proliferator activated receptors (PPARs) are a key family of ligand activated transcription factors that serve as crucial mediators in SCFA-induced regulation of metabolic syndrome (Kim et al., 2013; Den Besten et al., 2015). SCFAs stimulate secretion of the satiety hormones glucagon-like peptide-1 and peptide YY (PYY) in a GPR41- and GPR43-dependent manner and increase leptin levels in adipose tissue, thereby reducing food intake and weight gain (Tolhurst et al., 2012; Lu et al., 2016; Larraufie et al., 2018). PPARγ is predominantly expressed in the adipose tissue, and in mice with adipose-specific PPARγ destruction, SCFA-induced weight loss and insulin sensitivity stimulation disappeared (Den Besten et al., 2015; Yip et al., 2021). In a mouse model of asthma with GPR43 deficiency, the beneficial therapeutic effects of SCFAs on inflammation were lost (Maslowski et al., 2009). Higher levels of butyrate and propionate in stool samples from 1-year-old humans are associated with reduced atopic sensitization in children and a reduced likelihood of asthma at 3–6 years of age, indicating that SCFAs affect a child's susceptibility to allergic diseases (Roduit et al., 2019). Treatment with vancomycin reduced the decreased levels of SCFAs in mice, making them more susceptible to OVA-induced asthma, and supplementing exogenous SCFAs could alleviate this effect (Cait et al., 2018). This evidence supports the idea that SCFAs produced by fermentation of the gut microbiota may be a significant factor in obesity-related asthma susceptibility (Figure 2).

### 3.2. Chronic low-grade inflammation

#### 3.2.1. Obesity-associated chronic low-grade inflammation impacts asthma pathophysiology

Obese individuals exhibit characteristics of systemic chronic low-grade inflammation driven by relative hypoperfusion or increased oxygen consumption and sustained by leptin, leading to systemic monocyte activation (Lee et al., 2014; Reyes-Angel et al., 2022). Monocyte chemoattractant protein 1, secreted by the adipose tissue, binds to the monocyte surface receptor C-C chemokine receptor type 2 to promote monocyte activation and recruitment into the adipose tissue to form macrophages (Yao et al., 2022). Clinical studies revealed an inverse correlation between circulating monocytes in children with obesity-related asthma and low high-density lipoprotein levels along with significantly increased levels of soluble CD163, a measure of macrophage activation (Periyalil et al., 2015; Rastogi et al., 2015).

Macrophages directly sense pathogens through the expression of Toll-like receptors (TLR) and nucleotide-binding oligomerization domain-like receptors (NLR) (Sharma et al., 2018). FAs enhance TLR activation signals and are associated with the onset and progression of adolescent metabolic syndrome and asthma (Hardy et al., 2013; Zuo et al., 2015; Rocha et al., 2016; Meghnem et al., 2022). TLR recognition ligands activate various adaptor proteins downstream of myeloid differentiation factor 88 (MyD88)-dependent or non-MyD88-dependent pathways, initiating an inflammatory cascade that leads to the activation of nuclear factor-kappa B (NF-κB), resulting in an increased release of interleukin (IL)-6, tumor necrosis factor-α (TNF-α), and IL-1β (Kawai and Akira, 2010; Jialal et al., 2014). Lipopolysaccharide (LPS) is a classical TLR ligand. Whether LPS exposure is a protective or aggravating factor against asthma remains controversial. The role of LPS in airway inflammation has been observed in children with neutrophil asthma, and it induces macrophage inflammatory responses in mice (Camargo et al., 2018; Ciesielska et al., 2022). However, other studies suggested that the protective effect of the “farm effect” on asthma is specifically associated with LPS exposure. Importantly, this protective effect has only been observed during infancy (Schuijs et al., 2015; Gao et al., 2021).

NLRP3 is an important member of the NLR family (Wang and Hauenstein, 2020). NLRP3 expression is upregulated in response to TLR, activated by phosphorylation and deubiquitination, and then activated by stimuli such as porotoxins, leading to subsequent oligomerization, and inflammasome assembly (Song and Li, 2018). The assembled NLRP3 inflammasome cleaves pro-Caspase-1 proteolysis into mature Caspase-1 to promote the release of the inflammatory factors IL-1β and IL-18, mediating immune imbalances in asthma (Huang et al., 2021). NLRP3 inflammasome activation is a key phenotypic feature of obesity-related asthma, and *NLRP3* gene expression in the sputum of patients with obesity-related asthma is significantly increased and correlated with BMI (Wood et al., 2019).

#### 3.2.2. Gut microbiota influences asthma inflammation: the significance of LPS and NLRP3

A significant quantity of LPS accumulates in the intestine and enters the circulatory system by attaching to newly synthesized chylomicrons in the intestinal cell epithelium or increasing intestinal permeability, stimulating the immune response, and activating the TLR signaling pathway (Ghoshal et al., 2009; Velasquez, 2018). A host's LPS levels are influenced by the gut microbiota. Proinflammatory bacteria such as *Proteobacteria* carry Gram-negative LPS, and the HFD mice exhibited an increase in *Proteobacteria* abundance along with elevated levels of LPS (Mujico et al., 2013). Antibiotic intervention significantly reduces in LPS levels in the gut and circulation of HFD and ob/ob mice (Cani et al., 2008). Additionally, supplementation with *Bifidobacterium* reduced mouse intestinal LPS levels and improved gut barrier function (Cani et al., 2007). The gut microbiota stimulates the mucosal epithelial cells to release secretory immunoglobulin (Ig) A, mucin 2, and β-defensin, which are crucial for maintaining the intestinal mucosal barrier, reducing LPS translocation, and alleviating lung inflammatory damage (Dicks et al., 2018). Mouse experiments demonstrated that the gut microbiota activates the lung TLR4/NF-κB signaling pathway via the lung intestinal axis, aggravating LPS-induced acute lung injury (ALI) and that fecal microbiota transplantation can restore intestinal microbiotal homeostasis, increase intestinal flora diversity, and inhibit LPS-induced ALI (Tang et al., 2021). A cohort study reported that the immune response of asthmatic 17q21 risk allele carriers to LPS is regulated by the gut microbiota (Illi et al., 2022).

The gut microbiota plays a crucial role in mediating NLRP3 activation and inflammatory damage (Pellegrini et al., 2020; Pan et al., 2022). El Tor *Vibrio cholerae* triggers the NLRP3-dependent pathway, which induces IL-1β-mediated inflammatory responses that drive mouse macrophage death (BMDMs) (Mamantopoulos et al., 2019). In addition to specific species of gut microbes, he Rho GTPase activator CNF1, from *Escherichia coli* (*E. coli*) activates NLRP3 in BMDMs and leads to caspase-1 cleavage and IL-1β (Dufies et al., 2021). Probiotics present in the gut inhibit inflammasome expression. For instance, *Lactobacillus rhamnosus* GR-1 effectively reduces the expression of NLRP3 inflammatory bodies and caspase-1 induced by *E. Coli*, thereby limiting the occurrence of harmful inflammatory responses (Wu et al., 2016b). In an inflammatory bowel disease mouse model, it was discovered that NLRP3 mediated lung neutrophilic infiltrative inflammation in microbial pattern recognition, leading to increased levels of TNF and IL-1β levels in murine lungs (Liu et al., 2019). Furthermore, the gut microbiota exacerbated OVA-induced allergic asthma through the NLRP3/IL-1β signaling pathway in asthma model mice (Huang et al., 2018a; Zheng et al., 2022) (Figure 2).

### 3.3. Immune dysregulation

#### 3.3.1. Obesity-related immune dysregulation influences the onset of asthma

CD4^+^ T cells play a central role in the pathogenesis of asthma. Activated CD4^+^ T cells are divided into two subsets—T regulatory (Treg) and T effector (Teff) cells (Th1/Th2/Th17) with the former playing an immune regulatory role and the latter driving asthma pathogenesis and determining the asthmatic phenotype (Zhu et al., 2009). Under the influence of obesity, children with asthma exhibit a tendency for Teff cells to polarize toward Th1 and Th17 profiles. Multiple cytokines secreted by Th1 and Th17 cells mediate the development of neutrophilic asthma and are associated with asthma severity and steroid resistance (Nyambuya et al., 2020; Sze et al., 2020). Teff differentiation depends on the FAS pathway (Berod et al., 2014). Th17 cells polarization is boosted by increased *ACC1* gene expression and retinoic acid receptor related orphan receptor-γ t (RORγ t) binding to the *IL-17* gene locus (Zhang et al., 2021). Additionally, Th1 polarization in obesity-related asthma is influenced by macrophage activation and correlated with IL-6 and leptin levels (Reyes-Angel et al., 2022). Monocytes produce large amounts of IL-1β, which mediates Th17 cell differentiation (Revu et al., 2018). Obesity and atopic immunity are not mutually exclusive (Reyes-Angel et al., 2022). In obese children and adolescents, Th2-type asthma is associated with increased eosinophil infiltration and activity (Grotta et al., 2013). Furthermore, increased IgE levels and eosinophilic activation have been observed in obese mice (Amorim et al., 2018; Cvejoska-Cholakovska et al., 2019; Ying et al., 2022). This phenomenon is positively correlated with serum leptin and TNF-α levels (Grotta et al., 2013). Additionally, lipid mediators prostaglandin D_2_ and cysteinyl leukotriene can also activate Th2 cells and enhance the production of Th2 cell cytokines (Xue et al., 2015).

Innate lymphoid cells (ILCs) are innate T lymphocytes that express a profile of effector cytokines similar to those of T cells, enhance T cell function, and play a crucial role in asthma progression (Vivier et al., 2018). High ILC3 cell counts and RORC mRNA expression have been observed in the peripheral blood circulation of children with obesity-related asthma (Wu et al., 2018). In the lungs of obesity-related asthma mice, the NLRP3-IL-1β pathway is activated to induce the expansion of lung IL-17^+^ILC3 cells, leading to neutrophilic inflammation (Kim et al., 2014). In obese mice with AHR, ILC2 counts are increased, acting as Th2 cells but producing 10 times more IL-5 and IL-13 than activated Th2 cells (Everaere et al., 2016; Chen et al., 2017). The proliferation and function of ILC2 are influenced by lipid metabolism. Lipid droplets provide an energy source for pathogenic ILC2 responses during airway inflammation (Karagiannis et al., 2020). FAO and leptin play roles in driving ILC2 proliferation and maintaining their function (Wilhelm et al., 2016; Zheng et al., 2017). Under the chemotactic influence of lipids and inflammation, ILCs migrate within and between organs (Soriani et al., 2018). For example, sphingosine-1-phosphate mediates the migration of ILC2 to different tissues, thereby promoting the accumulation of ILC2 in lymphoid tissues, the bloodstream, and the lungs (Huang et al., 2018c).

#### 3.3.2. Gut microbiota modulates asthma immune response: focusing on CD4^+^ T cell and ILCs

A series of studies on antibiotic-treated and GF mice support the role of the gut microbiota in influencing T cell differentiation. Antibiotic-treated mice showed elevated levels of Th2 cytokines and IgE (Bashir et al., 2004). GF mice exhibit a loss of Th17 cells in the intestinal lamina propria and are more likely to produce a Th2 response (Wu et al., 2010; Herbst et al., 2011). A recent single-cell transcriptome study revealed that gut Teff are shaped by the microbiota independent of the typical subgroup regulators, T-bet, GATA3, or RORγt (Kiner et al., 2021). Several microbes, such as *Akkermansia muciniphila, Citrobacter rodentium*, and *Fusobacteriu varium*, induce T cell differentiation (Geva-Zatorsky et al., 2017; Stockinger, 2021; Liu et al., 2022). The influence of the microbiota on immune regulation can be transmitted to the offspring through the mother's gut microbiota and metabolites, thus accelerating the postpartum transition of the offspring from a Th2-dominated immunophenotype to Th1- and Th17-dominated immunophenotypes (Gao et al., 2021). The microbiota colonizing in the gut crosstalk pulmonary immunity via the gut lung axis, influencing host atopy and asthma (Pascal et al., 2018). CD4^+^ T cell dysfunction caused by dysregulation of the gut microbiota has been observed in newborns and is associated with susceptibility to allergic asthma in childhood (Fujimura et al., 2016). Segmented filamentous bacteria trigger a strong Th17-cell response in the gut and are preferentially recruited to the lungs to trigger immune inflammation (Bradley et al., 2017; Wang et al., 2019). *Ruminiclostridium* 6 and *Candidatus Arthromitus* mediate Th1/Th2 and Treg/Th17 immune balance in eosinophilic asthma in mice, suggesting that gut microbes regulate the balance between Teff subsets and participate in the pathogenesis of asthma (Zhou et al., 2022).

The response of ILCs to the gut microbiota is highly heterogeneous. ILCs expression are suppressed by microbial signal deficiency resulting from antibiotic treatment, with a greater impact observed on the gene expression profiles of ILC1 and ILC2 than ILC3 (Gury-BenAri et al., 2016). *Clostridioides difficile* infection upregulates the expressions of ILC1 and ILC3 in the colon, whereas *Helicobacter typhlonius* and *Helicobacter apodemus* infections lead to the ILC3 loss in the colon (Abt et al., 2015; Bostick et al., 2019; Kong et al., 2021). SCFAs activate ILCs via GPR signaling, promote ILC3 proliferation and IL-22 production, and inhibit ILC2 amplification (Yang et al., 2020; Sepahi et al., 2021). Furthermore, the gut microbiota promotes ILC3 production in the intestinal mucosa by assisting mononuclear phagocytes in secreting IL-1β and facilitating crosstalk between colony-stimulating factor 2 and RORγt+ cells (Mortha et al., 2014). The increase in intestinal ILC3 in the offspring of GF female mice after the implantation of *E. coli* HA 107 during pregnancy suggests that ILC formation by gut microbiota can be transmitted from the parents to the offspring (Gomez de Agüero et al., 2016). The gut microbiota regulates ILCs through the gut lung axis and contribute to airway inflammation and asthma. A previous study reported that *Proteobacteria* may promote the accumulation of natural ILC2 in the lungs by regulating the IL-33-CXCL16-CXCR6 signaling axis and interfering with the lung immune response (Pu et al., 2021). In a mouse model of asthma sensitized to house dust mites and characterized by gut dysbiosis attributed to *Candida* spp., there was an increase in lung ILC2 content, which resulted in exacerbated allergic airway inflammation and worsened disease control (Kanj et al., 2023). It suggests that gut microbiota imbalance may affect asthma symptoms through the regulation of ILC2 pathways (Figure 2).

## 4. Conclusion

Asthma is a complex disease with various phenotypes and endotypes, and related research should be focused on its well-defined classifications. The pathological basis of asthma in obese children is unique and involves multiple pathways, and the gut microbiota plays a pivotal role. Numerous clinical studies and basic experiments have confirmed the presence of gut microbiota dysbiosis in both obesity and asthma, indirectly indicating the involvement of the gut microbiota in the high-risk pathogenesis of obesity-related asthma. However, relevant clinical studies are lacking that explore the characteristics of gut microbiota dysbiosis in obese asthma patients, including the overall changes and exploration of specific strains. Therefore, the specific mechanisms by which alterations in the gut microbiota due to obesity lead to asthma have not yet been fully elucidated.

This article discusses various factors that influence the colonization and development of the gut microbiota, emphasizing the significant impact of early-life microbial dysbiosis on the susceptibility and progression of allergic and metabolic diseases in children. Furthermore, we elaborated on the role of the gut microbiota in regulating lipid metabolism, chronic inflammatory states, and immune responses, highlighting the potential key role of ecological imbalance in the pathogenesis of obesity-related asthma. This study's findings suggest that modulation of the gut microbiota could serve as an early therapeutic and preventive target for diseases such as asthma and obesity. However, clinical research on the characteristics of gut microbiota dysbiosis in obesity-related asthma, including overall changes and specific bacterial strains, remains scarce. Whether modulating the gut microbiota can be used as an early treatment and prevention target for diseases such as obesity, asthma, and obesity-related asthma requires extensive clinical and basic research. Relevant animal models must be refined to closely simulate human clinical conditions with particular attention paid to incorporating diverse age groups and their specific physiological and pathological backgrounds.

## Author contributions

MH: Writing—original draft, Writing—review & editing. XZ: Writing—original draft. YL: Writing—review & editing. HZ: Writing—review & editing. YY: Writing—review & editing. ZX: Writing—review & editing, Conceptualization, Funding acquisition, Supervision.
